# Where Have All the Parasites Gone? Modelling Early Malaria Parasite Sequestration Dynamics

**DOI:** 10.1371/journal.pone.0055961

**Published:** 2013-02-18

**Authors:** Deborah Cromer, Shannon E. Best, Christian Engwerda, Ashraful Haque, Miles Davenport

**Affiliations:** 1 Complex Systems in Biology Group, Centre for Vascular Research, University of New South Wales, Kensington, New South Wales, Australia; 2 Immunology and Infection Laboratory, Queensland Institute of Medical Research, Herston, Brisbane, Queensland, Australia; Instituto de Ciências Biomédicas/Universidade de São Paulo - USP, Brazil

## Abstract

Traditional approaches to measuring the level of malaria infection involve counting the proportion of parasite-infected red blood cells (iRBC) in circulating blood, known as parasitaemia. However, iRBC can also accumulate within the microvasculature of tissues and organs, a process called sequestration. Thus measurements of parasitemia do not necessarily reflect the total parasite burden (TPB). Recent experimental advances have allowed TPB measurements to be made in humans and experimental models. TPB is particularly important because it is the best current predictor of malaria disease severity and death in humans. Understanding the relationship between freely circulating iRBC versus tissue-sequestered iRBC is an important question in infection dynamics. The recent ability to experimentally measure the dynamics of iRBC in blood and tissue during murine malaria provides an exciting potential window into sequestration, but new modeling approaches are clearly required to understand these interactions. We present a model of malaria dynamics during early infection that incorporates iRBC that both circulate in the blood and sequester in tissue microvasculature. We explore the effect that perturbations to the system have on the ratio of the number of iRBC between these compartments, and consider which changes are most consistent with experimental data from mice. Using this model we predict an increase in the clearance rate of sequestered iRBCs around the time when mild symptoms become apparent, but a more pronounced increase in the rate of sequestration of iRBCs associated with the onset of severe malaria symptoms.

## Introduction

The term “severe malaria” encompasses a wide spectrum of syndromes, including severe anaemia, hyper-parasitaemia, acute respiratory distress, clinical jaundice, and cerebral malaria (CM) [1]. Severe malaria syndromes account for ∼900,000 deaths annually, with the majority of these caused by CM [1]. CM has been strongly associated with the packing of *Plasmodium-*infected red blood cells (iRBC) within, and obstruction of, brain microvasculature [2], [3]. However, the pathophysiology of CM is not fully understood, and may indeed differ significantly between individual cases, for example in adults versus children, and between different cohorts of children. Nevertheless, a general feature of severe malaria syndromes including CM is that the estimated total number of iRBC in the body (or Total Parasite Burden: TPB) is significantly higher than in patients suffering uncomplicated malaria [4]. In contrast, parasitaemia, a measure of iRBC circulating in the bloodstream is less reliable at differentiating patients with severe and uncomplicated malaria [4]. One hypothesis drawn from these observations in humans is that iRBC within host tissues play an important role in mediating severe disease symptoms during malaria.

The pathophysiology of severe malaria syndromes is difficult to study in humans. *In vivo* models of severe malaria are well-established, and may be useful for determining which factors control TPB, and how high TPB drives severe disease in humans. Infection of C57BL/6 mice with *P. berghei* ANKA (PbA), often referred to as “Experimental Cerebral Malaria (ECM)” elicits in mice several of the spectrum of symptoms that define severe malaria in humans, including metabolic acidosis, acute respiratory distress, liver dysfunction and neurological damage. Thus ECM may be considered as a model of severe malaria. During ECM, iRBC accumulate in multiple tissues, including the lung, liver, spleen, adipose tissue and brain [5], [6]. Given that both high TPB and inflammatory processes play a crucial role in ECM pathophysiology, this experimental system may be useful for modeling certain aspects of severe malaria in humans [7], [8], [9], [10].

Recent advances in genetic manipulation have allowed for the development of transgenic *Plasmodium* parasite lines that constitutively express bio-markers such as luciferase and GFP [11]. Coupled with *in-vivo* imaging techniques, these biomarkers permit the estimation of TPB *in vivo* and have clearly demonstrated the importance of TPB in driving ECM [5], [9], [12], [13]. In humans, estimates of TPB are possible through the measurement of parasite-derived proteins [4]. Despite the correlation between sequestered iRBC and malaria disease severity [14] and the demonstrated growth advantage associated with parasite sequestration [15], the causes of parasitised cell accumulation within tissues and the reasons for this growth advantage to the parasite remain the subject of conjecture [15].

A major difficulty in understanding the link between TPB and circulating parasitaemia is the dynamic nature of the relationship between the two. For example, parasitaemia could drop either due to increased tissue sequestration, increased splenic clearance, or a combination of the two. Mathematical modeling has been used for over 20 years to study the dynamics of iRBC circulating in the bloodstream, and has contributed to our understanding of red cell destruction, parasite preference and the role of innate and acquired immunity [16], [17], [18]. However, mathematical modelling of the dynamics of iRBC sequestration has seldom been conducted [19], [20].Although models relating PfHRP2 or circulating parasitaemia to total parasite numbers have been proposed [4], [21], and are important for interpreting the data at hand, these models are difficult to understand intuitively, and require assumptions to be made concerning parasite multiplication rate, and timing of parasitised cell sequestration.

We present a model of malaria infection that incorporates iRBC both in the blood and the tissue and considers the interplay between these two compartments during the first week of infection, when there is little adaptive immunity or resource limitation affecting parasite replication. Our model is both simple and intuitive. We explore the qualitative features of this model and consider how changes to the model parameters (which may arise in the normal course of infection) affect disease dynamics. It is particularly important to understand how such a model behaves qualitatively, as such understanding can often provide valuable insight into the changes that are occurring without requiring detailed model fitting. We show how our model can be applied to experimental data from mice in a simple manner and use it to develop two novel hypotheses about the clearance and sequestration rates of iRBC.

## Materials and Methods

### Sequestration Model

**Figure 1:**
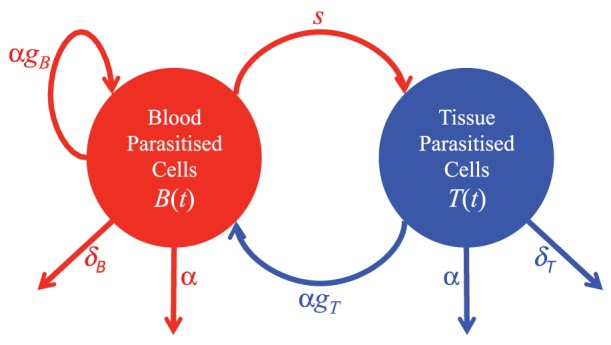
Proposed model of iRBC dynamics in the blood and tissue. The model consists of two parasitised cell populations: *B*(*t*), iRBC in the blood and *T*(*t*), iRBC in the tissue. Parasitised cells rupture at rate *α*/day, and parasitise new cells at rate *g_B_*/day if they rupture in in the blood compartment and rate *g_T_*/day if they rupture in the tissue compartment. All newly parasitised cells enter the blood compartment (i.e. they contribute to *B*(*t*)). Clearance of iRBC in the blood occurs at rate *δ_B_*/day and clearance in the tissue at rate *δ_T_*/day. Parasitised cells in the blood sequester into the tissue at rate *s*/day. *TPB*(*t*) (the total number of iRBC) is the sum of the number of iRBC in the blood and tissue.



We set up a continuous time model of parasitised cell dynamics in the blood and tissue. The model consists of two cell populations, iRBC in the blood, *B*(*t*) and iRBC that are sequestered in tissues, *T*(*t*). The total number of parasitised cells (total parasite burden, TPB(*t*)) is the sum of the number of iRBC in the blood and tissue. The model is depicted graphically in Figure 1 and described in equations *1a*–*1c* below.

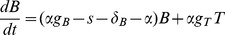
(1a)

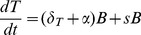
(1b)

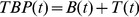
(1c)


Parasitised cells live for on average 1/*α* days, provided they are not destroyed prior to that time. Clearance of iRBC in the blood compartment occurs at a rate *δ_B_*/day and clearance of those in the tissue at rate *δ_T_*/day. Newly parasitised cells are generated as a result of rupturing iRBC from the blood and tissue at rates *g_B_* and *g_T_* respectively (where *g_B_* and *g_T_* are dimensionless quantities). All newly produced iRBC enter the blood compartment. Therefore the parasite multiplication rate (the number of newly generated infected cells per rupturing cell) is given by 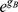 for cells rupturing from the blood and 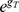 for cells rupturing from the tissue. This difference could arise as a result of cells in the tissue being in contact with more (or less) uninfected cells, and so they could give rise to a greater (or lesser) number of infected cells. Parasitised cells in the blood sequester into the tissue at a rate *s*/day.

Our model will be applied to the ECM system, which leads to severe symptoms approximately six days post infection. During this early stage of infection neither target cell limitation nor adaptive immunity is likely to significantly impact on disease dynamics, and therefore we have not included unparasitised red blood cells (RBCs) or immune cells in this model. An innate immune response is implicitly included via the parameters, *δ_B_* and *δ_T_*, which allow for the clearance of iRBC. We note that iRBC rupture at a rate that gives them an average lifetime of 24 hours (in the absence of other clearance mechanisms) rather than at a fixed time after invasion. Such a simplification is routinely used in models of malaria [22], [23], [24] and is justified in this case as no synchronization parasite rupture occurs in this experimental system. This allows our model to be solved analytically.

### Animals and Infection

#### Mice

Female C57BL/6 mice aged 6–8 weeks were purchased from the Australian Resource Centre (Canning Vale, Perth, Western Australia) and maintained under conventional conditions.

#### Ethics statement

All animal procedures were approved and monitored by the Queensland Institute of Medical Research Animal Ethics Committee. This work was conducted under QIMR animal ethics approval number A02–633M, in accordance with the “Australian code of practice for the care and use of animals for scientific purposes” (Australian National Health & Medical Research Council).

#### Parasites and infections

*P. berghei* ANKA (*Pb*A) strains were used in all experiments after one *in vivo* passage in mice. A transgenic *Pb*A (231c1l) clonal parasite line expressing luciferase and green fluorescent protein under the control of the EF1-α promoter (*PbA-*luc) was used for all experiments [25]. All mice were infected with 10^5^ pRBCs intravenously (i.v.) via the lateral tail vein. Blood parasitaemia was monitored by examination of Diff-Quick (Lab Aids, Narrabeen, NSW, Australia) stained thin blood smears obtained from tail bleeds.

#### *In vivo* bioluminescence imaging

Luciferase-expressing *Pb*A pRBCs were visualized by imaging whole bodies with an I-CCD photon-counting video camera and *in vivo* imaging system (IVIS 100; Xenogen, Alameda, CA). Mice were anesthetized with isofluorane and injected intraperitoneally with 0.1 ml of 5 mg/ml D-luciferin firefly potassium salt (Xenogen). 5 minutes afterwards, images were captured on the IVIS 100 according to the manufacturer’s instructions. Bioluminescence generated by luciferase transgenic *Pb*A in mice was measured according to the manufacturer’s instructions. The unit of measurement was photons/second/cm^2^/steer radiant (p/sec/cm^2^/sr). The *Pb*A line used in the animal experiments constitutively expresses luciferase throughout the parasite lifecycle [6]. Therefore the bioluminescence imaging quantifies the number of parasites present in an animal. Since the vast majority of infected cells contain only one parasite during *PbA* infection [26] (see Figure S1) we interpret the bioluminescence readings, expressed in units of photons/second/cm^2^/steer radiant (p/sec/cm^2^/sr), as being proportional the total number of iRBC present in an infected animal. All measurements were made at the same time each day.

## Results

### Parasitised Cell Growth during Experimental P. berghei ANKA Infection

**Figure 2:**
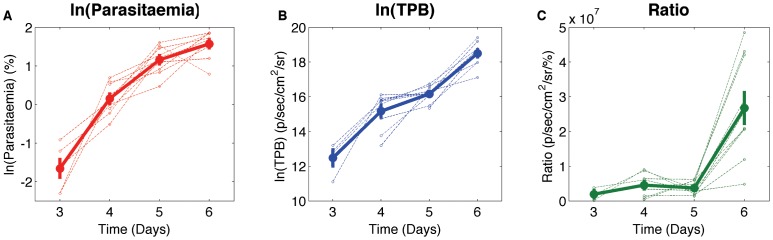
Experimental data of a *P. berghei* infection in 10 mice. (A) Parasitaemia (%) (B) TPB (p/sec/cm^2^/sr) (C) The ratio between TPB and parasitaemia (TPB/parasitaemia) (p/sec/cm^2^/sr/%). Thick solid lines show mean ± SEM of measurements from 10 mice. Thin dashed lines show measurements from individual mice.

The course of *PbA* infection is shown in Figure 2. The figure depicts the natural log of the parasitaemia, measured in % and corresponding to ln(*B*(*t*)) (panel A), the natural log of TPB, measured in p/sec/cm^2^/sr and corresponding to ln(*TPB*(*t*)) (panel B) and the ratio between TPB and parasitaemia, measured in p/sec/cm^2^/sr/% (panel C).

**Figure 3:**
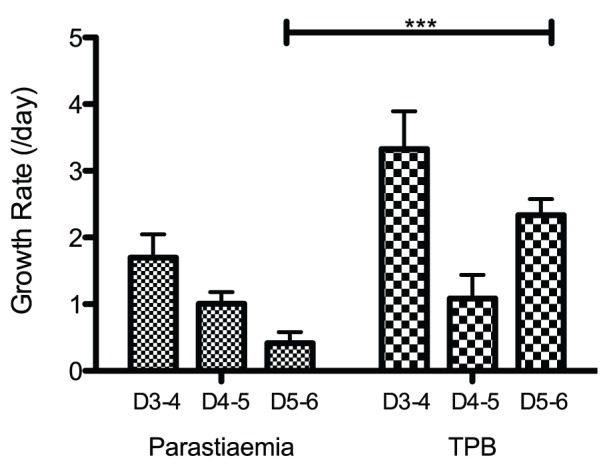
Comparison of Parasitaemia and TPB growth rates in the 10 mice. Growth rates (/day) for Parasitaemia and TPB from day 3 to day 4, day 4 to day 5 and day 5 to day 6. ***p<0.001.

Neither the growth rate of parasitaemia or TPB is constant over the course of the infection (Figure 2 and Figure 3). The growth rate of parasitaemia slows throughout the infection, while the growth rate of TPB slows between day 4 and 5, causing a transient decrease in the ratio between TPB and parasitaemia (Figure 2C) but then increases again between days 5 and 6, causing a large increase in the ratio. There is no significant difference between the growth rate of parasitaemia and that of TPB from day 3 to 4 or from day 4 to day 5 (*p*>0.19), however there is a significant difference between the growth rate of parasitaemia and that of TPB from day 5 to 6 (*p* = 6×10^−6^).

These differences in the growth and dynamics of parasites are consistent across multiple animals, and indeed across multiple independent experiments performed. They likely reflect changes in the host environment or host response to infection over this time. The goal of this work is to explore the iRBC dynamics predicted by the model described in Figure 1, and consider whether these dynamics are compatible with the observed data in Figure 2 and Figure 3.

### Understanding Sequestration Dynamics in a Simple Model

For constant parameter values we can solve the model in equations *1a* and *1b* analytically and derive expressions for *B*(*t*), the number of iRBC in the blood compartment and *T*(*t*), the number of iRBC in the tissue compartment. *TPB*(*t*), the total number of iRBC, then can be determined as the sum of *B*(*t*) and *T*(*t*) (equation *1c*). We introduce three summary parameters, *K*, *C* and *β* (defined in equations *2*–*4* below) to make future notation simpler. We let:
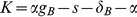
(2)

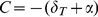
(3)and



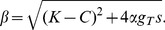
(4)Equations *1a* and *1b* can then be re-written as:
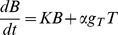
(5a)

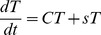
(5b)


And solved to give expressions for *B*(*t*) and *T*(*t*) as:
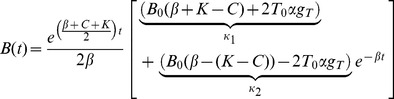
(6a)

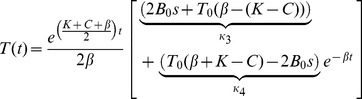
(6b)Where *B_0_* and *T_0_* are the starting numbers of iRBC in the blood and tissue respectively and the constants *κ_1−4_* depend only on the constant parameters from equations *1a* and *1b* and on the starting numbers of iRBC in the blood and tissue.

We see that provided enough time has elapsed since the start of infection (i.e. *t*>>*1/β*) the dominant growth rate of iRBC in both the blood and tissue (and hence of *TPB*(*t*)) is given by *γ*, where 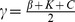. For biologically reasonable parameters this occurs whenever *t* is greater than ∼ 1 day (since *α*  = 1, *s* ≥1 *g_T_* ≥1.5 and hence necessarily *1/β* ≤1/6). Heuristically this can be understood by the fact that there is an inherent feedback between iRBC in the blood and tissue. Parasitised cells from the blood sequester into the tissue and, on average less than a day later, these cells rupture in the tissue, releasing merozoites that parasitise RBCs in the blood. Therefore, any change that affects the growth rate in either the blood or tissue compartment must eventually be felt in the other compartment, and the overall growth of iRBC in each compartment equalises. For the growth rate of either *B*(*t*) or *T*(*t*) to change, and to differ from each other, at least one of the model parameters must change. In the following sections we will consider possible parameter variations that result in different growth rates of *B*(*t*) and *T*(*t*).

Measurement of total parasite load using *in vivo* bioluminescence imaging gives a reading that is proportional to the total number of iRBC present in an animal, and therefore we will not measure exact numbers for *TPB*(*t*) experimentally. We therefore introduce a new quantity, *R*, the ratio between the measured TPB and circulating iRBC, *R* = *TPB*(*t*)/*B*(*t*). By considering the ratio of TPB and circulating iRBC we can provide insight into the dynamics without needing to know the exact number of iRBC in an animal. Equations *6a* and *b* and *1c* lead to the following expression for *R*:
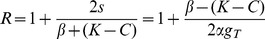
(7)


This expression does not depend on the starting numbers of iRBC in either compartment (See Appendix S1 for derivation of the expression for *R*).

### Is the Experimental Data Consistent with a Model that has Constant Parameter Values?

As we have previously noted, neither parasitaemia nor TPB is likely to be increasing at a constant rate for the entire infection and the growth rates of parasitaemia and TPB are not identical to each other throughout this experimental infection. Therefore the conclusions derived above do not hold, i.e. the growth rate of iRBC in the blood and whole body is not given by a constant value, *γ*, and therefore the experimental data is not consistent with a model having constant parameters values. At least one of the parameters of the model must be changing during the course of infection.

To establish the parameter changes that are most consistent with the experimental data in Figure 2 we consider the qualitative effect of altering each of the model parameters on: i) the observed growth rates of iRBC and ii) the observed ratio between TPB and circulating iRBC. We will compare the model outputs to the experimental data presented to determine which parameter changes are most likely to have occurred.

### Establishing the Effects of Parameter Variations on the Model Output

We simulate an injection of *P* parasitised cells into the blood compartment of a naïve animal i.e. *B*(0) = *P* and *T*(0) = 0. We allow the system to reach its equilibrium growth rate, *γ*, and then introduce a step change in each of the model parameters at time *t_1_*. We analyse the qualitative effect of each of these changes on TPB, circulating iRBC and on the ratio, *R*.

Expressions for *B*(*t*) and *T*(*t*) before and after the step change are shown in equations *8a* and *b* below, and we use these and equations *1c* and *7* to determine expressions for *TPB*(*t*) and *R* respectively. 
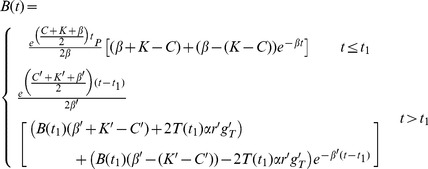
(8a)

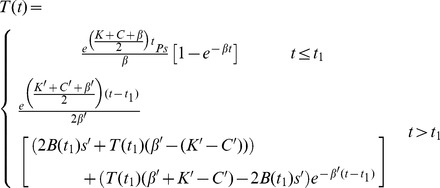
(8b)


Here *K*, *C*, and *β* denote the combination of parameters defined in equations *2*–*4* prior to the step change, and *K’*, *C’*, *β’* represent the same combinations after the step change. Similarly, individual parameters prior to the change are represented by their normal symbol, and parameters after the change are denoted with a dash.

**Figure 4:**
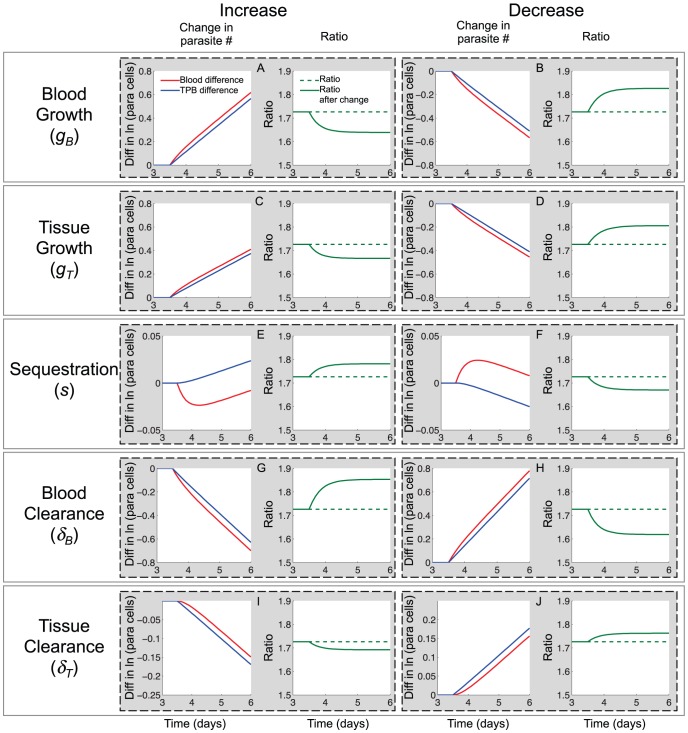
Effects of including a step change in each parameter at *t* = 3.5 days. The panels of the figure are annotated to show which parameter was changed, and the direction in which it was changed. All parameter changes occurred at 3.5 days after the parasitised cells were “injected”. Panels with red and blue lines show the difference in the log of the expected and observed numbers of circulating iRBC (red) and TPB (blue). Expected numbers are those that would have been observed in if all parameters had remained constant. Observed numbers are those observed after a parameter change. Panels with the green lines show the ratio, *R*, between the *TPB*(*t*) and *B*(*t*). In these panels the dotted line shows the expected ratio (constant parameters) and the solid line shows the observed ratio (after a parameter change). Note that both circulating iRBC and TPB grow at an identical rate before the parameter change (red and blue lines as well as dashed and solid green lines are overlaid for *t*<3.5 days) and also within approximately one day of the parameter change being made (red and blue lines are parallel, and green lines are flat for *t*>4.5 days). The y-axis scales differ between panels. This is because each parameter change has a different quantitative effect on the number of iRBC present in the blood and tissue. We are predominantly concerned with the qualitative effects of each parameter change and so can ignore these differences in scale.

We use equations *8a* and *b*, *1c* and *7* to determine the qualitative effect of each parameter change and show these in Table 1. These qualitative effects are independent of the actual parameter values, and depend only on the direction of the step change in a parameter (unless specified in Table 1). Figure 4 shows a graphical example of the effect of each parameter step change using two different representations: (i) The difference between the *expected* log of the number of iRBC and the *observed* log of the number of iRBC for both *B*(*t*) (red) and *TPB*(*t*) (blue) and (ii) the *expected* (dashed) and *observed* (solid) ratio, *R*. Note that *expected* values are those that would have been recorded *without* introducing a step change in the parameters and *observed* values are those that were observed after the parameter change. The baseline and perturbed parameter values are shown in Table 2.

**Table 1:** Summary of the qualitative effect of changes to individual parameters.

Parameter	Description	Change	Effect on circulating iRBC *B*(*t*)	Effect on Total Parasite Burden *TPB*(*t*)	Effect on Ratio *R* = *TPB*(*t*)/*B*(*t*)
*g_B_*	Growth in blood	↑	Growth rate ↑ Changes before *TPB*(*t*) does	Growth rate ↑ Changes after *B*(*t*) does	↓
		↓	Growth rate ↓ Changes before *TPB*(*t*) does	Growth rate ↓ Changes after *B*(*t*) does	↑
*g_T_*	Growth in tissue	↑	Growth rate ↑ Changes before *TPB*(*t*) does+	Growth rate ↑ Changes after *B*(*t*) does+	↓+
		↓	Growth rate ↓ Changes before *TPB*(*t*) does+	Growth rate ↓ Changes after *B*(*t*) does+	↑+
*s*	Sequestration	↑	Decreases growth rate of *B*(*t*) initially,followed by an overall increasein growth rate*	Increases growth rate of *TPB*(*t*) *	↑*
		↓	Increases growth rate of *B*(*t*) initially,followed by an overall reduced increasein growth rate*	Decreases growth rate of *TPB*(*t*) *	↓*
*δ_B_*	Killing in blood	↑	Growth rate ↓ Changes before *TPB*(*t*) does	Growth rate ↓ Changes after *B*(*t*) does	↑
		↓	Growth rate ↑ Changes before *TPB*(*t*) does	Growth rate ↑ Changes after *B*(*t*) does	↓
*δ_T_*	Killing in Tissue	↑	Growth rate ↓ Changes after *TPB*(*t*) does	Growth rate ↓ Changes before *B*(*t*) does	↓
		↓	Growth rate ↑ Changes after TPB(t) does	Growth rate ↑ Changes before *B*(*t*) does	↑
*δ_B_* and *δ_T_*	Overall Killing	↑	Growth rate ↓ If *δ_B_*-*δ_T_* is increased *B*(*t*)changes before *TPB*(*t*) if *δ_B_*-*δ_T_* isdecreased *B*(*t*) changes after TPB(t)	Growth rate ↓ If *δ_B_*-*δ_T_* is increased *TPB*(*t*) changes after *B*(*t*) if *δ_B_*-*δ_T_* isdecreased *TPB*(*t*) changesbefore *B*(*t*)	The effect on the ratio depends on the net effect on *δ_B_*-*δ_T_.* If the net effect is to
		↓	Growth rate ↑ If *δ_B_*-*δ_T_* is increased *B*(*t*)changes before *TPB*(*t*) if *δ_B_*-*δ_T_* isdecreased *B*(*t*) changes after *TPB*(*t*)	Growth rate ↑ If *δ_B_*-*δ_T_* is increased *TPB*(*t*) changes after *B*(*t*) if *δ_B_*-*δ_T_* isdecreased *TPB*(*t*) changesbefore *B*(*t*)	increase *δ_B_*-*δ_T_* the ratio ↑. If the net effect is to decrease *δ_B_*-*δ_T_* the ratio ↓.

+ Provided condition S.1 in Appendix S2 holds.

* Provided 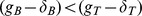, i.e. provided that sequestered iRBC have a growth advantage over non-sequestered iRBC [15] (see Appendix S2).

**Table 2:** Parameter values used to generate Figure 4.

Paramete (units)	Description	Baseline value	Increased Value	Decreased Value	Equivalent to
*g_B_* (dimensionless)	Growth rate in the blood	1.8	2.2	1.4	4–9 merozoites successfully invading following iRBC rupture [35]
*g_T_* (dimensionless)	Growth rate in the tissue	2	2.4	1.6	5–11 merozoites successfully invading following iRBC rupture [35]
*δ_B_* (/day)	Clearance rate of iRBCin the blood	0.6	1.1	0.1	Clearance rate of infected cells in blood ranges from 0.1–1.1/day
*δ_T_* (/day)	Clearance rate of iRBCin the tissue	0.2	0.35	0.05	Clearance rate of infected cells in tissue ranges from 0.05–0.35/day
*s* (/day)	Rate of sequestration from the blood to the tissue	1.2	1.3	1.1	iRBC sequester on average at between 18 and 22 hours [30], [36].
*α* (/day)	Rate at which iRBCrupture.	1	N/A	N/A	iIRBC rupture after an average time of 1 day [30].

Left-most column shows the baseline parameter values that were used in all simulations except the ones where the parameter was specifically listed as being changed. Values in the middle column are the parameters used for “increased” parameter values and values in the right-most column are values used for a “decreased” parameter value. Note that the value of *α* was not changed, as the length of iRBC lifetime is well established.

**Figure 5:**
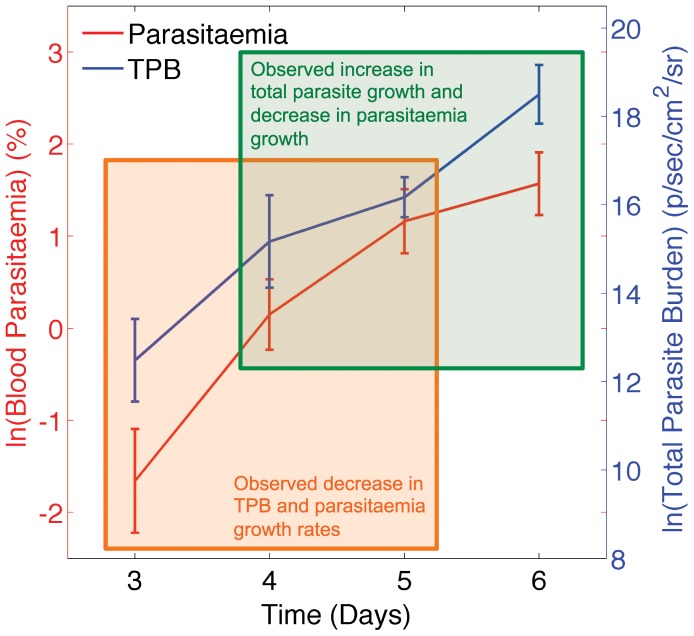
Data from showing mean ± SEM of circulating parasitaemia (red) and TPB (blue) in the 10 mice. The natural log of the experimental values are shown. Note that circulating parasitaemia (red) is plotted against the left-hand y-axis and is measured in units of %, while TPB (blue) is plotted against the right hand y-axis and measured in units of p/sec/cm^2^/sr. Units are shown on each axis. Orange shaded area highlights the data that is used in Figure 6, and indicates a decrease in the growth rate of both parasitaemia and TPB after day 4. Green shaded area highlights the data that is used in Figure 7 and indicates an increase in the growth rate of the TPB after day 5 coupled with a decrease in the growth rate of parasitaemia.

The changes described in Table 1 and Figure 5 assume that the model parameters change instantaneously, although in reality parameters are likely to increase or decrease over an extended time period. This would not alter the qualitative effects described in Table 1 and Figure 5 (Figure S2). Additionally, in reality there may be more than one parameter change occurring concurrently. If these have different effects on the growth rates and ratio, the combined effect would need to be evaluated using equations *8a* and *b*, *1c* and *7*.

### Insights Gained by Applying the Sequestration Model to Experimental Data

We now apply the qualitative understanding we have gained above to help interpret the experimental data in Figure 2 without directly fitting of the model to the data. We infer which parameters in the model are most likely to have changed based on the qualitative changes that are observed in the experimental data and use this as an illustrative example of the benefits that can be derived from a thorough understanding of the model underlying the experimental data.

Using the growth rates depicted in Figure 3, we observe a potential change in iRBC growth rates at two time points – either after day 4 or after day 5. After day 4 the growth rate of circulating iRBC decreases (*p* = 0.41) as does the growth rate of TPB (*p* = 0.042). After day 5 the growth rate of TPB increases (*p* = 0.017) and the growth rate of circulating iRBC continues to decrease (*p* = 0.096). We apply two separate example analyses to the experimental data to interpret these different periods: (i) We assume that both circulating iRBC and TPB are growing at a constant rate, *γ_1_*, between day 3 and 4 and ask what parameter change could have occurred after day 4 to generate the iRBC levels observed on day 5; and (ii) We make an alternative assumption, that both circulating iRBC and TPB were growing at a constant rate, *γ_2_*, between day 4 and 5 and ask what parameter change could have occurred after day 5 to generate the iRBC levels observed on day 6. We note that since no significant difference between the growth rate of circulating iRBC and TPB was observed either between days 3 and 4 or between days 4 and 5 (Figure 3) these assumptions of constant growth are valid.

#### Example 1 - What may have caused the observed decrease in growth rate of iRBC?

We first focus on the orange shaded area of Figure 4. Between day 3 and day 4 growth rates of circulating iRBC and TPB are not significantly different from each other (Figure 3, *p* = 0.19). We estimate the growth rate of iRBC in the blood and tissue using data from the 10 mice and equation *9* below.
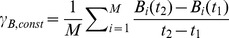
(9a)

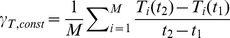
(9b)


Within equations *9a* and *b*, *γ_Β_,_const_* and *γ_Τ_,_const_* is the constant growth rate we are estimating for parasitaemia and TPB respectively, *i* is the mouse index, *t_1_* and *t_2_* are the times at the start and end of the interval over which we are assuming constant growth and *M* is the number of mice with parasitaemia (or TPB) measurements recorded at both *t_1_* and *t_2_*.

**Figure 6:**
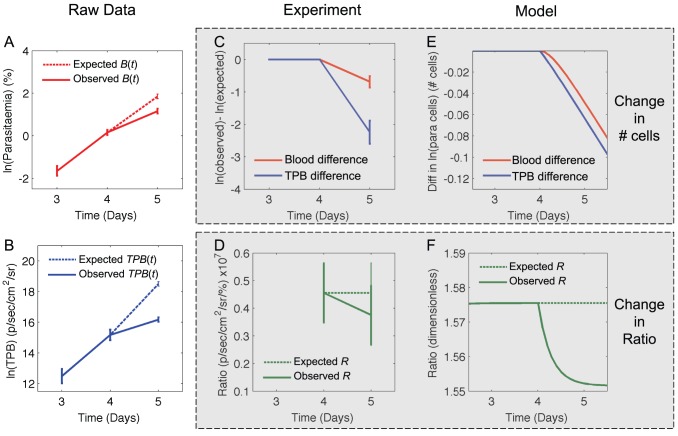
Explanation of potential parameter changes after day 4. (A and B) Log of the expected (dashed lines) and observed (solid lines) values for parasitaemia (A) or TPB (B). Data shows mean ± SEM of measurements from 10 mice. Expected values are calculated by assuming that the growth rates of parasitaemia and TPB are constant and equal to *γ_1_* (defined in the text) between day 4 and day 5. (C) Difference between the log of the expected and the log of the observed values of parasitaemia (red) and TPB (blue) if cells grew at constant rate *γ_1_*. (D) The ratio between TPB and blood parasitaemia. Dashed line shows the expected ratio (if iRBC continue to grow at a constant rate, *γ_1_*, between day 4 and day 5) and solid line shows the observed ratio. (E and F) Output of a model simulation with an increase in the rate of clearance of sequestered iRBC after day 4. Parameters before day 4 are: *g_B_* = 1.8, *g_T_* = 2.8, *s* = 1.1, *δ_B_* = .6, *δ_T_* = .2. After day 4, *δ_T_* = .35. Panel E shows the difference between the log of the expected and observed values if *B*(*t*) (red) and *TPB*(*t*) (blue). Note that although panels C and E and panels D and F appear qualitatively similar, the units between them are different, as the experimental and simulated data have very different units. Panels C and D present *B*(*t*) in units of % of cells *TPB*(*t*) in units of p/sec/cm^2^/sr and *R* in units of p/sec/cm^2^/sr/%, while panels E and F present *B*(*t*) and *TPB*(*t*) in terms of number of cells and *R* as a dimensionless quantity.

We use *γ_Β_,_const_* and *γ_Τ_,_const_* to project the average parasitaemia and TPB respectively forward from day 4 to day 5. This determines the expected average parasitaemia and TPB on day 5 if no parameter change occurred. We compare the log of these expected values to the log of the observed parasitaemia and TPB values on day 5 (Figure 6A and B). Both the observed parasitaemia and TPB on day 5 are lower than would be expected.

We next generate plots resembling those in Figure 5 that show (i) the difference between the log of the observed and expected numbers of iRBC (Figure 6C) and (ii) the ratio between TPB and the circulating iRBC (Figure 6D) and determine the panels of Figure 5 to which these plots are most similar. Figure 6C and D are most similar to panel I of Figure 5 indicating that the experimental data are most consistent with an increase in the clearance rate of sequestered iRBC between day 4 and day 5. Plots of a model simulation showing an increase in this parameter, *δ_T_*, at *t* = 4 days are shown in panels E and F of Figure 6 for comparison with the experimental data in panels C and D. Note that the y-axis scales of Figure 6C and D are very different to those of Figure 5 and Figure 6E and F as different units have been used. Therefore we only make qualitative comparisons between the panels.

Importantly we could not have drawn the conclusion that this data was most consistent with an increase in the clearance rate of sequestered iRBC from the experimental data alone. Without an understanding of the underlying model we could not have determined whether a decrease in parasite multiplication rate, an increase in splenic clearance or an increase in the clearance rate of sequestered iRBC was most likely to be responsible for the observed decrease in the growth rates of iRBC. Only by understanding the effects of each parameter change on both parasitaemia and TPB and on the ratio, and by comparing these to the experimental data can we determine that an increase in the clearance rate of sequestered iRBC is most consistent with the experimental data.

#### Example 2 - What may have caused the observed increase in the TPB growth rate?

**Figure 7:**
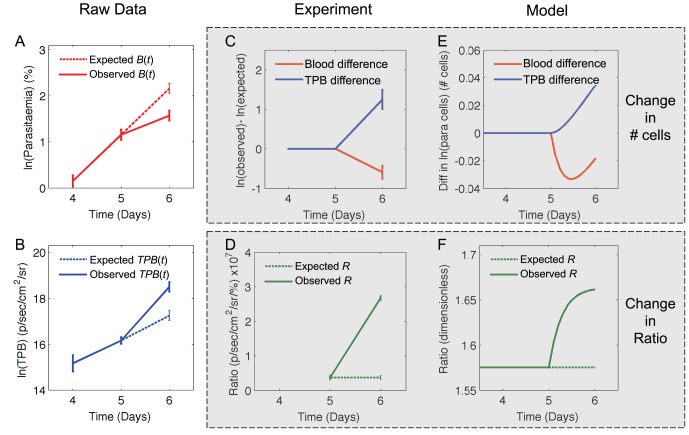
Explanation of potential parameter changes after day 5. (A and B) Log of the expected (dashed lines) and observed (solid lines) values for parasitaemia (A) or TPB (B). Data shows mean ± SEM of measurements from 10 mice. Expected values are calculated by assuming that the growth rates of parasitaemia and TPB are constant and equal to *γ_2_* (defined in the text) between day 5 and day 6. (C) Difference between the log of the expected and the log of the observed values of parasitaemia (red) and TPB (blue) if cells grew at constant rate *γ_2_*. (D) The ratio between TPB and blood parasitaemia. Dashed line shows the expected ratio (if iRBC continue to grow at a constant rate, *γ_1_*, between day 4 and day 5) and solid line shows the observed ratio. (E and F) Output of a model simulation with an increase in the rate of sequestration after day 5. Parameters before day 5 are: *g_B_* = 1.8, *g_T_* = 2.8, *s* = 1.1, *δ_B_* = .6, *δ_T_* = .2. After day 5, *s* = 1.3. Panel E shows the difference between the log of the expected and observed values if *B*(*t*) (red) and *TPB*(*t*) (blue). Note that although panels C and E and panels D and F appear qualitatively similar, the units between them are different, as the experimental and simulated data have very different units. Panels C and D present *B*(*t*) in units of % of cells *TPB*(*t*) in units of p/sec/cm^2^/sr and *R* in units of p/sec/cm^2^/sr/%, while panels E and F present *B*(*t*) and *TPB*(*t*) in terms of number of cells and *R* as a dimensionless quantity.

We next apply the same analysis to the data shown in the green shaded area of Figure 4. We assume the model parameters are constant between 4 and day 5 (this assumption is reasonable as the red and blue lines are approximately parallel over this time) and that both circulating iRBC and TPB are growing at a constant rate *γ_2_*, once again determined using equations *9a* and *b*. We calculate the expected and observed parasitaemia and TPB on day 6 as described above, and show these in Figure 7A and B, along with the difference between observed and expected numbers (Figure 7C) and the observed and expected ratios (Figure 7D). Once we again compare these to the panels of Figure 5. We find that Figure 7C and D are most similar to panel E in Figure 5, indicating that the data are consistent with an increase in the rate of sequestration, *s* after day 5. Model output from a simulation with an increase in the value of *s* at *t* = 5 days is shown in panels E and F of Figure 7 for comparison.

## Discussion

Recent advances in experimental techniques have provided methods to measure the TPB of *Plasmodium-*infected humans and experimental animals, which supplement established routine methods for measuring parasitemia. TPB measurements provide much needed information on the numbers of iRBC outside of the circulating bloodstream during a malaria infection, and have led to a number of new hypotheses regarding the mechanisms of iRBC sequestration [4], [5], [21], [27].

Mathematical models have been useful in helping to interpret the dynamics of circulating iRBC [23], [24], [28], [29]. Some previous work has also attempted to model sequestration in patients under drug therapy, but these reports considered circulating and not tissue sequestered iRBC [19], [20]. Indeed, there has been little attempt to incorporate new TPB data into novel or existing mathematical models beyond attempts to quantify the exact numbers of sequestered parasites [4], [21]. Additionally, there has been little analysis of the effect that perturbations to the system can have on iRBC dynamics.

In this paper we present a model of iRBC growth and accumulation in the blood and tissue. In the absence of any perturbations to the system iRBC in the blood and tissue grow at identical rates and the ratio between TPB and circulating iRBCs remains constant. Perturbations to the model parameters (either as a result of an increase in cytokine production [5], increased splenic clearance or other immune responses to infection), result in qualitative changes in the growth rates of iRBC in each compartment. We have developed an understanding of how such parameter changes impact on the model dynamics and have used our to predict that clearance of sequestered iRBCs increases at the time of onset of mild symptoms (day 4–5 in this experimental system) and that the rate that iRBC sequester into the tissue increases at the time of severe malaria symptoms and just prior to the animal succumbing to infection (from day 5 to day 6 in this system).

The model presented in this paper, while being relatively simple still has 6 parameters that govern its behaviour. It is highly likely that some or all of these parameters vary over the course of an infection due to host and parasite responses and therefore fitting this model to experimental data without either some prior insight into the underlying model dynamics or an extremely large dataset is likely to yield parameter estimates with very large confidence intervals. However, once a better understanding of the model is obtained, it is possible to draw conclusions about how the system may be changing even without making exact parameter estimates. By considering changes in the growth rate of parasitaemia and TPB we have generated two novel hypotheses that present new avenues for future research and have resulted directly from our modelling approach. We predict that the clearance rate of iRBC in the tissue increases after day 4 of infection. Future experimentation is now needed to determine whether clearance of iRBC in tissue does indeed vary through the course of infection. Secondly, we predict that sequestration of iRBC increases after day 5 of infection. Although the parallels between murine and human malaria remain a subject of debate, the general consensus is that when appropriately employed, these models can inform upon human disease [30], [31].

The work presented in this paper marks an important step in applying mathematical models to data on TPB and iRBC sequestration during malaria infection. Such understanding has previously been gained for models describing circulating iRBC during malaria [23], [24], [28], as well as in HIV [32], [33], [34] and has proved to be a necessary foundation for future work. It is only with a solid understanding of the dynamics underpinning iRBC in blood and tissue that we can probe more deeply into the complexities of parasite sequestration. This work has generated novel hypotheses, which may ultimately help others prevent the onset of severe malaria in *Plasmodium-*infected humans.

## Supporting Information

Figure S1 **Histogram showing the number of parasites found inside each iRBC on day 5 of infection.** Data shows results from 5 mice and is representative of 5 of independent experiments performed.
(EPS)

Figure S2 **Effects of including a change in each parameter at** ***t*** **= 3.5 days, where the change takes place over an entire day.** The panels of the figure are annotated to show which parameter was changed, and the direction in which it was changed. All parameter changes began at 3.5 days after the parasitised cells were “injected” and continued until *t* = 4.5days. The quantitative value of the changes are identical to those in Figure 2, however the change takes place over one day, rather than instantaneously. Panels with red and blue lines show the difference in the log of the expected and observed numbers of circulating iRBC (red) and TPB (blue). Expected numbers are those that would have been observed in if all parameters had remained constant. Observed numbers are those observed after a parameter change. Panels with the green lines show the ratio, R, between the *TPB*(*t*) and *B*(*t*). In these panels the dotted line shows the expected ratio (constant parameters) and the solid line shows the observed ratio (after a parameter change). Note that both circulating iRBC and TPB grow at an identical rate before the parameter change (red and blue lines as well as dashed and solid green lines are overlaid for *t*<3.5 days) and also within approximately one day of the parameter change being made (red and blue lines are parallel, and green lines are flat for *t*>4.5 days). The y-axis scales differ between panels. This is because each parameter change has a different quantitative effect on the number of iRBC present in the blood and tissue. We are predominantly concerned with the qualitative effects of each parameter change and so can ignore these differences in scale.
(EPS)

Appendix S1 **Deriving an expression for the Ratio, R.**
(DOCX)

Appendix S2 **Derivation of the requirement that sequestered iRBC must have a growth advantage over non-sequestered iRBC.**
(DOCX)
