# Supplementary material for: Where Have All the Parasites Gone? Modelling Early Malaria Parasite Sequestration Dynamics
Source: PLoS One. 2013 Feb 18;8(2):e55961. doi: 10.1371/journal.pone.0055961 (PMC3575381; doi:10.1371/journal.pone.0055961)
Supplement: Appendix S1 — Deriving an expression for the Ratio, R. (DOCX) [file pone.0055961.s003.docx]

# Appendix S1

### Deriving an expression for the Ratio, *R*

Equations *6a* and *b* and *1c* in the main paper lead to an expression for *R* as:

$$\begin{matrix} R & =1+\frac{\left(2B_{0}s+T_{0}(\beta-(K-C))\right)+\left(T_{0}(\beta+K-C)-2B_{0}s\right)e^{-\beta t}}{\left(B_{0}(\beta+K-C)+2T_{0}\alpha rg_{T}\right)+\left(B_{0}(\beta-(K-C))-2T_{0}\alpha rg_{T}\right)e^{-\beta t}} \\ & \begin{matrix} \Rightarrow1+\frac{2B_{0}s+T_{0}(\beta-(K-C))}{B_{0}(\beta+K-C)+2T_{0}\alpha rg_{T}}\text{ as }t\to\infty\text{, since }e^{-\beta t}\to0\text{ as }t\to\infty\\ =1+\frac{2s}{\beta+(K-C)} \\ =1+\frac{\beta-(K-C)}{2\alpha g_{T}} \end{matrix} \end{matrix}$$

Therefore *R* does not depend on the starting numbers of iRBC in either compartment.
