# Supplementary material for: Where Have All the Parasites Gone? Modelling Early Malaria Parasite Sequestration Dynamics
Source: PLoS One. 2013 Feb 18;8(2):e55961. doi: 10.1371/journal.pone.0055961 (PMC3575381; doi:10.1371/journal.pone.0055961)
Supplement: Appendix S2 — Derivation of the requirement that sequestered iRBC must have a growth advantage over non-sequestered iRBC. (DOCX) [file pone.0055961.s004.docx]

# Appendix S2

### Derivation of the requirement that sequestered iRBC must have a growth advantage over non-sequestered iRBC

Let *A* be the “advantage” a parasite has by sequestering, and define
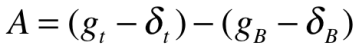
.

We first consider how the ratio, *R*, changes as the parameter g*_t_* changes. Let $Z=\beta+K-C$. Then when *Z* increases, *R* decreases, and visa-versa (provided that *s* remains constant). We can establish that:

$$\frac{dZ}{dg_{t}}=-(1-r)\left(\frac{\frac{2rs}{1-r}-(K-C)}{\beta}-1\right)$$

and therefore whenever

$g_{t}<\frac{rs}{1-r}+A+s$ S.1

the change in *R* is in the same direction as the change in *g_t_*.

We next consider how the equilibrium growth rate in blood and tissue, *γ*, changes as the parameters *r* and *s* change. We can establish that:

$\begin{matrix} \frac{d\gamma}{dr} & =-\frac{g_{t}}{2}\left(1-\frac{(g_{t}r+s)-A}{\beta}\right) \\ & =-\frac{g_{t}}{2}\left(1-\frac{(g_{t}r+s)-A}{\sqrt{\left((g_{t}r+s)-A\right)+4sA}}\right) \end{matrix}$. S.2

Using equation S.2 we see that when *A* < 0 then
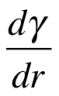
 > 0 and when *A* > 0 then
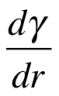
 < 0. A similar expression can be used to show that when *A* < 0 then $\frac{d\gamma}{ds}$ < 0 and when *A* > 0 then $\frac{d\gamma}{ds}$ > 0.
